# Polyphasic evaluation and cytotoxic investigation of isolated cyanobacteria with an emphasis on potent activities of a *Scytonema* strain

**DOI:** 10.3389/fmicb.2022.1025755

**Published:** 2022-10-28

**Authors:** Trang T. Ngo, Bich-Loan T. Nguyen, Tuan A. Duong, Thu-Huyen T. Nguyen, Thanh L. Nguyen, Kien T. Kieu, Minh-Hanh T. Do, Sang V. Nguyen, Nguyen Dinh Thang, Hang T. L. Pham

**Affiliations:** ^1^Faculty of Biology, University of Science, Vietnam National University, Hanoi, Vietnam; ^2^Department of Biochemistry, Genetics, and Microbiology, Forestry and Agricultural Biotechnology Institute, University of Pretoria, Pretoria, South Africa; ^3^Center for Life Sciences Research, Faculty of Biology, University of Science, Vietnam National University, Hanoi, Vietnam; ^4^The Key Laboratory of Enzyme and Protein Technology, University of Science, Vietnam National University, Hanoi, Vietnam

**Keywords:** *Aulosira*, *Desmonostoc*, *Desikacharya*, *Hapalosiphon*, *Scytonema*, polyphasic approach

## Abstract

Cyanobacteria are phototrophic organisms widely found in most types of natural habitats in the tropical regions of the world. In this study, we isolated and identified cyanobacterial strains from paddy soil in Hanoi (Vietnam) and investigated their cytotoxic activities. Five isolated cyanobacterial strains showed distinctive profiles of gene sequences (rRNA 16S and *rbcL*), phylogenetic placements, and morphological characteristics. Based on the polyphasic evaluation, they were classified as *Scytonema bilaspurense* NK13, *Hapalosiphon welwitschii* MD2411, *Aulosira* sp. XN1103, *Desikacharya* sp. NS2000, and *Desmonostoc* sp. NK1813. The cytotoxic screening revealed that the extract of strain *Scytonema bilaspurense* NK13 exhibited potent cytotoxic activities against four human cell lines of HeLa cells, OVCAR-8 cells, HaCaT cells, and HEK-293T cells, with IC_50_ values of 3.8, 34.2, 21.6, and 0.6 μg/mL, respectively. This is the first time a well-classified *Scytonema* strain from tropical habitat in Southeast Asia has been recognized as a potential producer of cytotoxic compounds.

## Introduction

Cyanobacteria are considered as a new and promising source of structurally diverse and pharmacologically interesting compounds (Qamar et al., 2021; Saeed et al., 2022). Over the past four decades, more than 260 metabolite families (with 1,630 unique compounds) have been isolated from cyanobacteria exhibiting 14 types of bioactivities, of which cytotoxic metabolite families account for 42 % (Demay et al., 2019). Many compounds from cyanobacteria, such as Apratoxin A, Tasipeptins A–B, Coibamide A, Largazole, Dolastatins, and Cryptophycins, served as lead structures for designing new cancer therapy and entering clinical trials. Notably, dolastatin derivatives had a therapeutic effect that was 100 folds higher than the anti-cancer drug vinblastine (Luesch et al., 2001) and was then developed into brentuximab vedotin 63; the drug has been approved by US Food and Drug Administration (FDA) and European Medicines Evaluation Agency (EMEA) for the treatment of leukemia since 2015 (Bajpai et al., 2018). Because of the increasing discovery rate of new structural compounds, cyanobacteria have been considered as an alternative medicinal source for traditional microbial drug producers like Actinomycetes and Hyphomycetes (Olaizola, 2003; Saeed et al., 2022).

Although cyanobacteria have been identified as a promising group of organisms from which new anti-cancer-type natural products have been discovered and developed (Mondal et al., 2020; Qamar et al., 2021), their taxonomic classification remains a problematic issue. During the period 1970–2000 s, the nomenclature of many cytotoxic–producing cyanobacterial strains was solely based on morphological characteristics, leading to the misclassification at the species/genus/family level. Demay et al. (2019) reported that it was difficult to determine whether several bioactive compounds were produced by *Lyngbya* or *Moorena* strains because many *Moorena* strains were previously morphologically placed in the *Lyngbya* genus. Therefore, the classification of cyanobacteria based on a polyphasic approach that primarily relies on the sequences of the selected marker genes is fundamental and a prerequisite for determining the biological source of lead structures in any biological activity studies.

Vietnam is characterized by an agricultural economy with 22% of land areas utilized for rice cultivation, which forms an enormous habitat for nitrogen-fixing cyanobacteria. Based on morphological characteristics, Duong (1994) reported that at least 18 genera of cyanobacteria had frequently been found in the paddy soil, including *Gloeocapsa, Chlorogloea, Plectonema, Lyngbya, Oscillatoria, Anabaena, Anabaenopsis, Aulosira, Nostoc, Cylindrospermum, Calothrix, Tolypothrix, Scytonema, Fischerella, Hapalosiphon, Mastigocladus, Stigonema*, and *Westiellopsis*. Most of the research on Vietnamese cyanobacteria thus has focused on exploiting the cyanobacteria of paddy soil for agriculture and pharmaceutical purpose. In a study by Pham et al. (2017), 26 extracts of 13 cyanobacterial strains isolated from paddy soil in Central Vietnam were screened for cytotoxic activities, indicating that three extracts exhibited cytotoxic activities on MCF7 and HCT116 cells with an IC_50_ ranging from 47.8 to 232.0 μg/mL. In another study, the Nostoc-like strain CAVN10 isolated from paddy soil in Northern Vietnam produced three carbamidocyclophanes (A–C) with strong cytotoxic activities against MCF7; the IC_50_ values were calculated at 0.68–2.2 μM (Bui et al., 2007). In order to extend the pharmaceutical investigation of cyanobacteria from different paddy soil locations in Vietnam, this study focused on the classification and cytotoxic activity evaluation of isolated cyanobacteria in the suburban region of Hanoi. The isolated strains were identified based on the polyphasic approach, which revealed a potent cytotoxic strain belonging to the *Scytonema* genus.

## Materials and methods

### Sampling sites

The paddy soil samples were collected from Dong Anh (between 21°10′N and 21°11′N latitude, and 105°49′E and 105°51′E longitude), Soc Son (21°24′N latitude and 105°82′E longitude), and Quoc Oai (21°00′N latitude and 105°37′E longitude) districts, Hanoi, Vietnam. At each study site, samples were taken at three different positions or trees from April to September 2019.

### Cyanobacteria isolation

Each soil sample (1g) was suspended in liquid BG11 medium; then, 0.5 mL of each sample was transferred onto a BG11 agar plate (Petri dish) using the streaking technique. All Petri dishes were incubated under a 12:12 light/dark cycle with white fluorescent irradiation (10 μmol m^−2^ s^−1^) for 2 weeks to grow cyanobacteria. Monoclonal cyanobacterial strains were purified from the primary isolation plates using the streak-plate procedure, and axenic strains were obtained as described in the previous study (Pham et al., 2017). All isolated strains were deposited in the cyanobacteria collection of the Faculty of Biology, University of Science, Hanoi, Vietnam.

### Morphological characterization

The morphological examination was carried out using a Zeiss Axioplan II Fluorescence Microscope with digital Camera BUC5F-2000 C, and a Zeiss Axio Observer equipped with Apotome 2 and AxioCam MRm camera. Images were taken from cultures obtained from the exponential phase to the stationary phase of the cyanobacterial life cycle. Cell dimensions of vegetative cells, heterocysts, and akinetes were measured from live materials as described by (Pham et al., 2017).

### DNA extraction, PCR amplification, and sequencing

The DNA was extracted from 2-week-old cultures, following the procedure described in a previous study (Pham et al., 2017). The crude extracted DNA solution was kept at - 20°C and used directly as templates in PCR reactions for amplifying 16S rRNA and *rbcL* genes by using specific primers:

Primer 1: 5′- CTC TGT GTG CCT AGG TAT CC - 3′.
Primer 2: 5′- GGG GGA TTT TCC GCA ATG GG - 3′.
Primer 6: 5′- GAC GGG CCG GTG TGT ACA - 3′.
rbcLF forward primer: 5′-GAC TTC ACC AAA GAY GAC GAA AAC AT-3′.
rbcLR reverse primer: 5′- GAA CTC GAA CTT RAT YTC TTT-3′.

A part of the 16S rRNA gene region was amplified through a nested PCR technique in which a pair of primer 1 and primer 2 (Boyer et al., 2001) was used in the first round of amplification, and a pair of primer 2 and primer 6 (Boyer et al., 2002) was used in the second round of PCR. The PCR reaction component and PCR thermal cycle were the same as in a previous study (Pham et al., 2017). The *rbcL* gene was amplified by using primers rbcLF and rbcLR (Singh et al., 2015). The PCR reaction mixture consisted of 15.0 μL of 2X PCR Master Mix (GoTaq^®^, Promega, US), 1.0 μL (0.3 μM) each of the forward and reverse primers, 1.0 μL of the extracted DNA solution, and PCR grade water to a final volume of 30 μL. The PCR thermal cycle consisted of an initial denaturation step at 95°C for 2 min, followed by 40 cycles of 95°C for 20 s, 56°C for 20 s, 72°C for 1 min 30 s, and a final extension step at 72°C for 10 min.

The PCR products were separated and visualized using 1% agarose gel electrophoresis (added 1 μL of RedSafe dye in 25 mL of agarose) for 25 min, at a voltage of 100 V. The size of DNA bands was then examined under ultraviolet (UV) light in comparison to a 1 kb DNA Ladder (Cleaver Scientific Ltd, UK). Finally, the amplicon products were sent to 1st BASE (Malaysia) for Sanger sequencing.

### Phylogenetic analyses

Consensus sequences of both 16S rRNA and *rbcL* genes were assembled from forward and reverse sequencing reads using the CLC Genomics Workbench v8.0.1 (CLCBio, Denmark). BLAST searches were conducted with all sequences obtained from this study against the GenBank nucleotide database, from which most closely related sequences were downloaded and included along with sequences of type species from cyanodb.cz (Hauer and Komarek, 2022) in the subsequent phylogenetic analyses. The *Synechococcales, Spirulinales, Pleurocapsales, Oscillatoriales, Gloeobacterales, Chroococcales*, and *Chroococcidiopsidales* served as outgroups. The datasets were aligned using MAFFT v7.221 (Katoh and Standley, 2013), trimmed at both ends, and thereafter subjected to Maximum Likelihood (ML) and Bayesian Inference (BI) analyses. ML analyses were conducted using IQ-TREE v2.1.2 (Bui et al., 2020) with best nucleotide subsection models automatically determined and an SH-like approximate likelihood ratio test (Guindon et al., 2010), as well as an ultrafast bootstrap (Bui et al., 2013; Hoang et al., 2018) of 1,000 replicates each. BI analyses were conducted using MrBayes v3.2.5 (Ronquist et al., 2012) using the same model as determined by ML analyses. Ten parallel runs, each with four chains, were carried out. Trees were sampled every 100th generation, and runs were set to auto-stop when the average standard deviation of split frequencies was 0.01 or lower. The burn-in fraction of 0.25 was discarded, and the posterior probability values were calculated from the remaining trees.

### Preparation of extracts

Each axenic strain was cultivated in a sterile BG11 medium without CO_2_ aeration, at 25 ± 2°C under a 12:12 light/dark cycle with white fluorescent irradiation (20 μmol m^−2^ s^−1^). All strains were manually shaken one time every 2 days. The cyanobacterial biomass was harvested after 7 weeks by centrifugation at 6,000 × *g* for 10 min at 20°C, followed by lyophilization. Freeze-dried microalgae cells were extracted with a mixture of organic solvent of 50% ethyl acetate and 50% methanol following the procedure described in a previous study with several modifications (Pham et al., 2017). First, 2 g of dried biomass from each strain was homogenized in 100 mL of organic solvents using a mortar and pestle, followed by sonication for 30 min and shaking for a further 30 min at room temperature. The homogenized solution was centrifuged at 5,000 × g for 10 min at 20° C, and the supernatant was collected by filtration. The residue was extracted two times with 100 mL of mixture solvent, and all supernatants were combined to obtain the crude extract. The organic solvents were removed by vacuum rotary evaporation. The amorphous solid extracts were weighed and stored at – 20° C until use.

### Cytotoxicity assays

The MTT (Sigma Chemical Co., St. Louis, MO, USA) kit was used for the evaluation of the cytotoxic activity of cyanobacterial extracts as described by Nguyen et al. (2021). Four human cell lines obtained from ATCC were used for this test, including human cervical adenocarcinoma cells (HeLa), human ovarian carcinoma cells (OVCAR-8), human epidermal keratinocyte cells (HaCaT), and human embryonic kidney cells (HEK-293T). Cells were seeded in a 96-well microplate with a density of 5,000 cells/well in 200 μL of culture medium (DMEM/RPMI 1640; Sigma Aldrich, USA) and cultivated at 37°C and 5% CO_2_ for 24 h. After that, each well was supplemented with 50 μL of cyanobacterial extracts at a final concentration ranging from 0.06 μg/mL to 200.0 μg/mL (for HeLa cells and HEK-293T cells) and from 3.125 μg/mL to 100.0 μg/mL (for OVCAR-8 cells and HaCaT cells). Paclitaxel (Sigma-Aldrich, USA) was used as a positive control at concentrations ranging from 0.1 nM to 125.89 nM. Each concentration was tested in four replicates, and the entire experiment was repeated 3 times. After 48 h of treatment, 200 μL of MTT solution (0.5 mg/mL) was added to each well and incubated for 4 h at 37°C. MTT solution was discarded, and then 100 μL of DMSO was added to dissolve the formed formazan. The optical density was measured using a Microplate Reader SpectraMax Plus^384^ (Molecular Devices, USA) at 570 nm and 690 nm wavelengths to detect the viable cells. The IC_50_ values (the concentration of extract at which 50% of cell growth is inhibited) were calculated by GraphPad Prism 8 software.

## Results and discussion

### Isolation and morphology characterization

**Figure 1:**
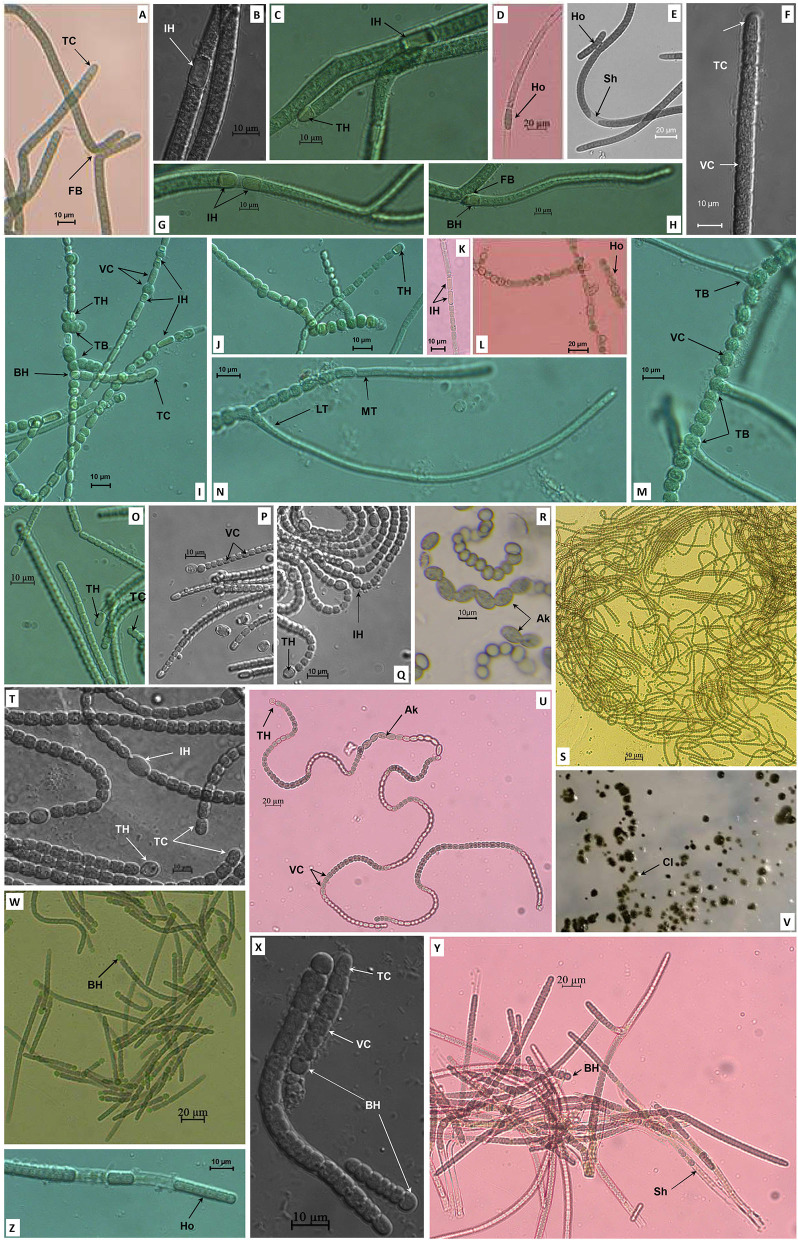
Morphology of strains NK13 **(A–H)**, MD2411 **(I–N)**, NS2000 **(O–Q)**, NK1813 **(R–V)**, and XN1103 **(W–Z)**. TB, True branching; FB, False branching; MT, Main trichome; LT, Lateral trichome; TH, Terminal heterocyst; IH, Intercalary heterocyst; BH, Basal heterocyst; Ak, Akinete; VC, Vegetative cell; TC, Terminal cell; Ho, Hormogonia; Sh, Sheath; Cl, Colony.

Five cyanobacterial strains were isolated from paddy soil with distinctive morphological and morphometrical characteristics, and were named as NK13, MD2411, NK1813, NS2000, and XN1103. Strain NK13 expressed typical two lateral branches (Figure 1A) of Scytonema-like strains as described by Komárek et al. (2013); sometimes, a single false branching occurred at the basal heterocyst (Figure 1H). The intercalary oblong heterocysts frequently appeared solitary (Figures 1B,C), and rarely in pairs (Figure 1G). The terminal heterocysts appeared when growing in the BG11_0_ (nitrogen-deficient BG11) medium. Trichomes were slightly curved with square to cylindrical (longer than wide) vegetative cells (Figures 1F,C) and attenuated terminal cells (Figures 1A,F) in a thin sheath (Figure 1E).

Strain MD2411 demonstrated true branching (Figures 1I,J,L–N), which could be observed in the members of *Hapalosiphon, Westiella*, and *Westiellopsis* genera of the Hapalosiphonaceae family (Mishra et al., 2013; Komárek et al., 2017). The branches usually developed from one side (Figure 1I), and rarely on both sides (Figure 1M). In the branches, the heterocysts were observed at the basal or second position (Figure 1I). Both main trichomes and branches contained intercalary and terminal heterocysts with hemispherical, spherical, or cylindrical shapes (Figures 1I–K), and cylindrical developing vegetative cells at the apical end (Figure 1N). Thus, the appearance of heterocystous branches [as described by Tan et al. (2016)] and the identical morphometric cells located in the main trichomes and branches [as described by Ferreira et al. (2013)] implied their resemblance to morphospecies of *Hapalosiphon* (Figures 1I,J,N).

Strain XN1103 possessed the gray square vegetative cells in a distinct sheath (Figures 1Y,Z). The morphological characteristics of trichomes were clearly altered through the life cycle. The subspherical greenish heterocysts appeared at the base of all young trichomes (Figures 1W,X) and were dehiscent in mature trichomes (Figure 1Y). Constriction at the cross wall was slight in the young trichomes (Figure 1X) and became ambiguous in the old trichome (Figure 1Y). The old trichomes were isopolar without any heterocysts. These morphological characteristics of the strain XN1103 were similar to those of the genera *Aulosira, Nodularia, Fortiea* (Komárek et al., 2017), and *Calochaete* (Hauer et al., 2013).

Two strains, NK1813 and NS2000, showed Nostoc-like morphology with long, coiled, and isopolar trichomes that were clearly constricted at the cross walls (Figures 1Q,S,U). The heterocysts appeared both at the terminal and intercalary positions (Figures 1Q,T); the terminal cells were not different from the vegetative cells (Figures 1O,P,T). Nevertheless, strain NK1813 was distinguished by the presence of a series of akinetes, up to 13 cells (Figures 1R,U), which was completely absent in strain NS2000. Furthermore, the colonies of strain NK1813 were round and covered with a fine mucilaginous sheath (Figure 1V). Besides, all the parameters of vegetative cells, terminal cells, and heterocysts of strain NK1813 were better than those of strain NS2000 (Table 1).

**Table 1:** Morphological and morphometrical characteristics of five isolated cyanobacterial strains.

**Strains**	**Trichome features**	**Vegetative cells**	**Terminal/apical cells**	**Heterocysts**	**Akinetes**	**Sheath**
MD2411	Dark blue-green, true-branching (T-type)	Ranged from barrel-shaped, spherical to cylindrical, clearly constricted at the cross walls, 3.3–7.7 μm wide and 3.3–11.7 μm long	Round or cylindrical with attenuated to the end, 3.3–5.0 μm wide and 5.8–7.5 μm long	Hemispherical, spherical, or cylindrical, 3.2 −5.0 μm wide and 3.0–11.1 μm long	Absent	Thin hyaline
NK13	Dark blue-green, isopolar or heteropolar, single or double false-branching	Not constricted at the cross walls; cylindrical to barrel shaped; width (5.2–7.1 μm) is usually more than length (5.5–6.7 μm) in old trichome but nearly square in young trichome.	Tapering, 5.1–6.7 μm wide and 5.4–8.5 μm long	Oblong with convex ends at intercalary (one or in pair) with 6.7–8.2 μm wide and 8.3–12.7 μm long; solitary at basal/terminal rounded conical.	Absent	Thin and colorless
NK1813	Olive green, isopolar, long and slight coiling	Barrel-shaped, oval to spherical, 5.0–7.1 μm wide and 5.2–7.2 μm long	Oblong curved at the ends, 4.9–6.6 μm wide and 6.6–7.8 μm long	Solitary at intercalary and terminal filament; intercalary heterocysts are elliptic (8.3–10.8 μm wide and 10.0–13.5 μm long), terminal heterocysts are elliptic to spherical (9.8–10.2 μm in diameter)	Appear in a series (2–13 cells), elliptic with 5.2–9.6 μm wide and 11.8–18.6 μm long	Thin hyaline
NS2000	Dark blue-green, isopolar, slight coiling	Square to cylindrical, 3.4–4.9 μm wide and 3.0–5.3 μm long	Cylindrical with curved end, 3.3–4.5 μm wide and 3.6–4.5 μm long	Solitary at terminal and intercalary filament, terminal heterocysts elliptic with 4.5–5.5 μm wide and 5.2–5.4 μm long; terminal heterocysts elliptic or attenuated (4.1–6.2 μm wide, 5.7 −7.3 μm long)	Absent	Thin diffluent
XN1103	Greenish–gray, young trichome heteropolar, older trichome isopolar	Cylindrical or square with 4.5–7.2 μm wide and 4.2–7.1 μm long	Rounded conical, 3.6–5.0 μm wide and 3.6–6.6 μm long	Basal, single, only in young older trichome, hemispherical to spherical, 5.5–6.6 μm wide and 5.0–7.6 μm long	Absent	Distinct, colorless, opens at the ends

In summary, the morphological characteristics of strains NK13 and MD2411 were similar to the members of two genera *Scytonema* and *Hapalosiphon*, respectively. Strains NK1813 and NS2000 resembled the morphological characteristics of *Nostoc*, while the strain XN1103 presented the traits of several genera, including *Aulosira, Nodularia*, and *Fortiea*. These strains developed hormogonia as reproductive propagules (Figures 1D,E,L,Z). The detailed morphometrical characteristics of heterocysts, akinetes, and vegetative terminal cells are listed in Table 1. For the classification of five isolated cyanobacteria, the 16S rRNA sequencing and *rbcL* gene sequence analysis were combined with morphological features in the polyphasic approach.

### Molecular barcode and phylogeny analysis

**Figure 2:**
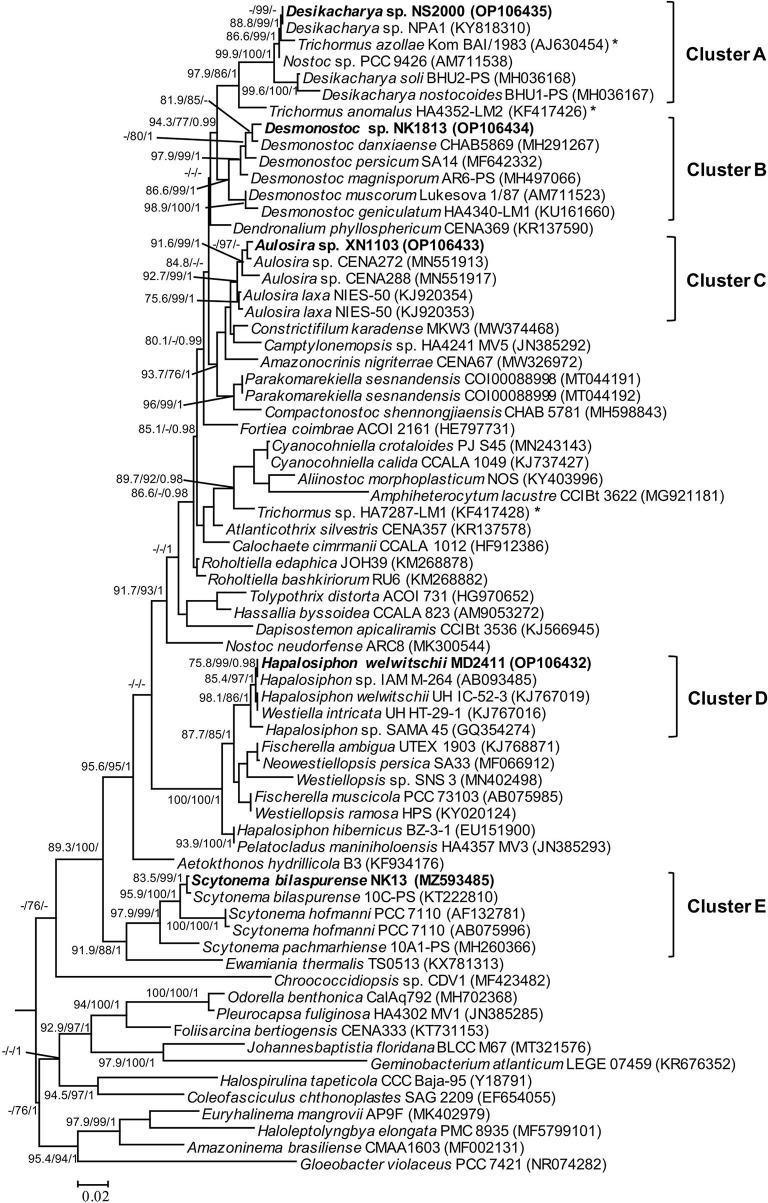
Phylogram derived from maximum likelihood (ML) analyses of 16S rRNA sequence dataset. The analyses were conducted for 10 independent runs using MrBayes v3.2.5, and each run was carried out for 5 million generations by applying the GRT+G+I substitution model. The likelihood ratio test (SH-aLRT) (%) and ultrafast bootstrap support (BS) (%) values of IQTREE >75% and Bayesian posterior probabilities (BPP) >0.95 are indicated at nodes as SH-aLRT/BS/BPP. Scale bar indicates estimated substitutions per site; *: marks for the position of the *Trichormus* strain showing a polyphyletic relationship.

The PCR amplification of 16S rRNA yielded expected products of about 900 bp in size. The 16S rRNA sequences from all strains were included together with 65 reference sequences from public databases to construct a phylogeny. The dataset also included recently described taxa in the order Nostocales, including *Amazonocrinis nigriterrae, Dendronalium phyllosphaericum, Constrictifilum karadense*, and *Atlanticothrix silvestris* (Alvarenga et al., 2021; Chavadar et al., 2021). Phylogenetic trees obtained from ML and BI analyses had similar topologies at the terminal clades. However, the tree from ML analysis revealed much better support for many of the nodes that were not supported in BI analysis. Five isolated strains from this study were assigned to five genera (corresponding to five clusters A-E in Figure 2) based on monophyletic clustering with the type strain of each genus coinciding with the species and genus threshold proposed by Kim et al. (2014) and Yarza et al. (2014). The 16S rRNA sequence similarity between the isolated strains and the closest reference strains from Genbank is displayed in Table 2. Besides, the morphological characteristics and habitat types were carefully validated for species and genus delimitation.

**Table 2:** Percentage identities of the 16S rRNA gene region between isolated strains described in this study and other most related strains from GenBank.

	**Cyanobacterial strains**	**1**	**2**	**3**	**4**	**5**	**6**	**7**	**8**	**9**	**10**	**11**	**12**	**13**	**14**	**15**	**16**	**17**
1.	**NK1813**																	
2.	*Desmonostoc danxiaense* CHAB5869	**99.28**																
3.	*Desmonostoc persicum* SA14	**99.15**	99.27															
4.	**MD2411**	92.23	92.36	91.95														
5.	*Hapalosiphon welwitschii* UH IC-52-3	92.23	92.36	91.95	**100**													
6.	**NK13**	91.29	91.02	91.17	90.17	90.17												
7.	*Scytonema bilaspurense* 10C-PS	91.01	90.74	90.89	90.17	90.17	**99.76**											
8.	*Scytonema pachmarhiense* 10A1 PS	90.05	89.91	89.64	89.38	89.38	95.40	95.14										
9.	*Scytonema* sp. BHUS-5	90.58	90.31	90.46	89.73	89.73	**99.15**	99.15	94.75									
10.	**NS2000**	94.74	94.74	94.49	91.85	91.85	92.41	92.40	90.63	91.98								
11.	*Nostoc* sp. NPA1	94.74	94.74	94.49	91.85	91.85	92.41	92.40	90.63	91.98	**100**							
12.	*Nostoc* sp. PCC 9426	94.99	95	94.75	92.11	92.11	92.14	92.14	90.90	91.72	**99.76**	99.76						
13.	*Nostoc neudorfense* ARC8	96.03	95.77	95.64	91.94	91.94	90.97	90.70	89.03	90.26	95.25	95.25	94.99					
14.	*Desikacharya soli* BHU2-PS	94.61	94.61	94.73	92.51	92.51	91.16	91.16	89.64	90.73	**98.04**	98.04	98.29	95.14				
15.	**XN1103**	95.80	96.31	96.69	93.14	93.14	91.45	91.18	89.51	90.75	94.89	94.89	95.14	94.99	94.46			
16.	*Aulosira laxa* NIES-50	96.05	96.55	96.81	92.74	92.74	90.92	90.64	89.81	90.21	95.39	95.39	95.65	94.73	94.85	**98.79**		
17.	*Aulosira* sp. CENA272	95.93	96.43	96.81	92.87	92.87	91.19	91.18	89.38	90.76	95.14	95.14	95.40	94.60	94.98	**99.52**	99.03	

The bold values indicate the percentage identities of the 16S rRNA gene region between isolated strain and reference strains within a genus. The total alignment length was 908 bp including gap positions.

**Figure 3:**
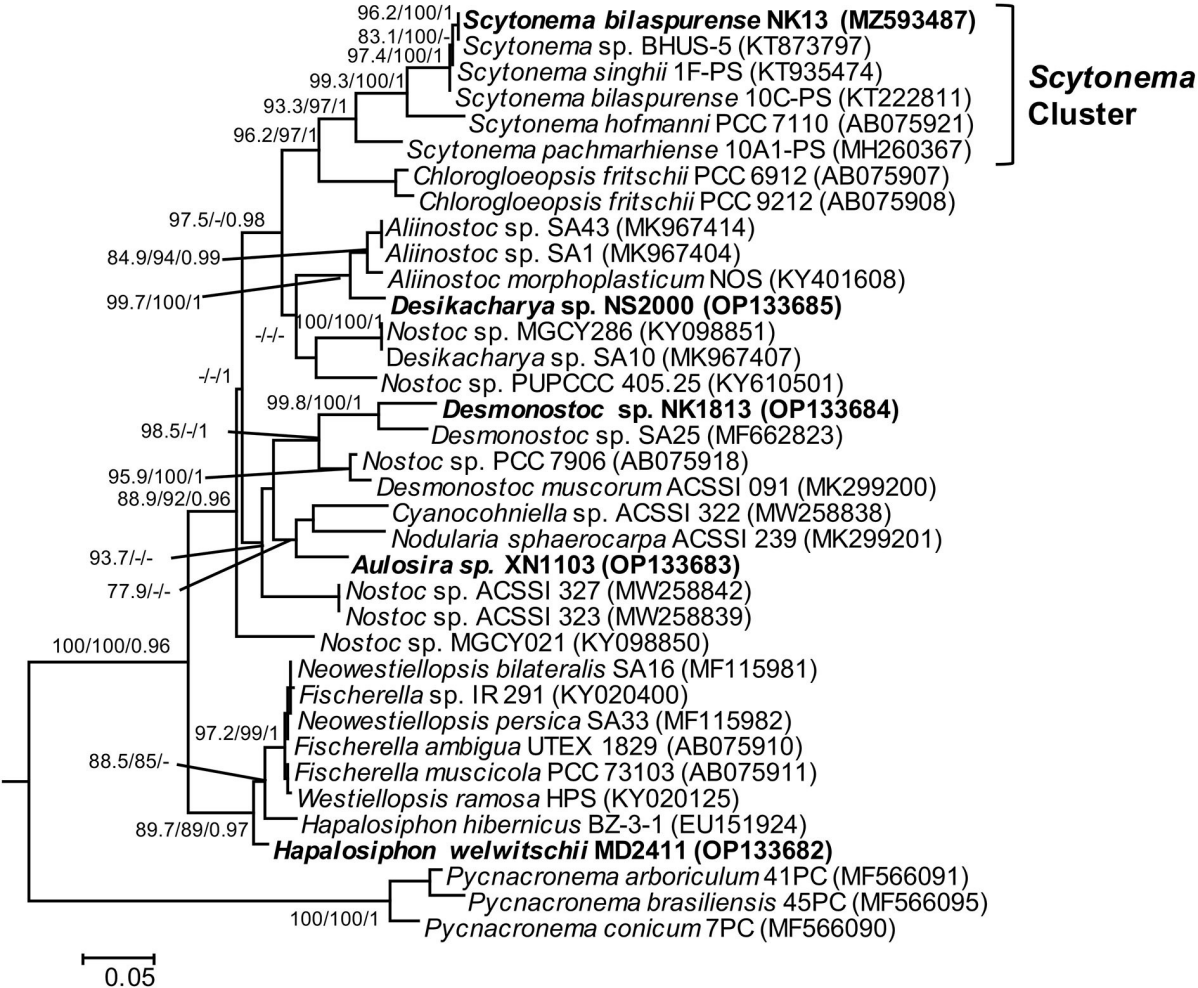
Phylogram derived from maximum likelihood (ML) analyses of *rbcL* sequence dataset. The analyses were conducted for 10 independent runs using MrBayes v3.2.5, and each run was carried out for 5 million generations by applying the GRT+G+I substitution model. The likelihood ratio test (SH-aLRT) (%) and ultrafast bootstrap support (BS) (%) values of IQTREE >75% and Bayesian posterior probabilities (BPP) >0.95 are indicated at nodes as SH-aLRT/BS/BPP. Scale bar indicates estimated substitutions per site.

Strain NK13 shared the highest 16S rRNA sequence similarity of 99.76 % with strain *Scytonema bilaspurense* 10C-PS (Table 2), and both strains clustered with the typical *Scytonema* strains, including *Scytonema hofmanni* PCC7110 and *Scytonema pachmarhiense* 10A1-PS (Komárek et al., 2013; Singh et al., 2016; Saraf et al., 2018), by a relatively high SH-aLRT/BS/BPP values of 97.9/99/1 (Figure 2, cluster E). The phylogenetic tree based on *rbcL* gene sequences further supported the placement of strain NK13 in the genus *Scytonema* (Figure 3), in which it shared 100% and 99.63% *rbcL* gene sequence similarity with *Scytonema* sp. BHUS-5 (KT873797) and *Scytonema bilaspurense* 10C-PS (KT222811), respectively (Table 3). Although strain NK13 showed an identical sequence of the *rbcL* gene, it shared only 99.15% sequence similarity of the 16S rRNA gene with strain *Scytonema* sp. BHUS-5 (Table 2). Thus, based on the most closet 16S rRNA gene sequence similarity and phylogenetic relatedness to *Scytonema bilaspurense* 10C-PS, strain NK13 was provisionally identified as *Scytonema bilaspurense*. In addition, the appearance of strain NK13 was in congruence with the description of *Scytonema bilaspurense* (Singh et al., 2016) by double/single false branching, shape, and dimension of vegetative cells/heterocysts. Therefore, based on polyphasic evaluations (morphology, molecular characterization, and freshwater habitat), strain NK13 was classified as *Scytonema bilaspurense*.

**Table 3:** Percentage identities of the *rbc*L gene region between isolated strains described in this study and other most related strains from GenBank.

	**Cyanobacterial strains**	**1**	**2**	**3**	**4**	**5**	**6**	**7**	**8**	**9**	**10**	**11**	**12**	**13**	**14**
1.	**MD2411**														
2.	*Hapalosiphon hibernicus* BZ 3 1	**95.66**													
3.	Westiellopsis ramosa HPS	96.43	95.68												
4.	**NK13**	85.25	83.91	84.57											
5.	*Scytonema* sp. BHUS-5	85.25	83.91	84.57	**100**										
6.	*Scytonema bilaspurense* 10C-PS	84.78	83.70	84.58	**99.63**	99.63									
7.	*Scytonema pachmarhiense* 10A1 PS	86.66	86.26	85.57	91.57	91.57	92								
8.	**NK1813**	84.09	82.98	84.10	89.62	89.62	90.06	89.87							
9.	*Desmonostoc* sp. SA25	83.72	82.55	83.32	86.59	86.59	87.05	87.99	**93.10**						
10.	**NS2000**	86.39	85.94	84.82	88.96	88.96	88.51	87.65	90.55	88.61					
11.	*Aliinostoc morphoplasticum* NOS	86.26	84.50	85.59	88.13	88.13	87.68	88.13	91.41	89.32	95.85				
12.	*Desikacharya* sp. SA10	86.62	85.80	85.52	88.12	88.12	88.13	88.55	86.80	85.54	**90.10**	90.74			
13.	**XN1103**	89.14	86.81	87.86	87.47	87.47	87.47	88.86	88.40	89.69	88.63	89.50	88.65		
14.	*Nodularia sphaerocarpa* ACSSI 239	86.45	85.12	85.51	85.82	85.82	85.83	88.09	86.78	85.72	88.30	88.94	87.65	92.89	

The bold values indicate the percentage identities of the rbcL gene region between isolated strain and reference strains within a genus. The total alignment length was 700 bp including gap positions.

The strain MD2411 resided in the same clade (Figure 2, cluster D) with three other *Hapalosiphon* strains (*Hapalosiphon welwitschii* UH IC-52-3, *Hapalosiphon* sp. IAM M-264, and *Hapalosiphon* sp. SAMA 45) with SH-aLRT/BS/BPP value of 98.1/86/1. Of these, *Hapalosiphon welwitschii* UH IC-52-3 was also isolated from the soil sample in Australia (Stratmann et al., 1994), showing similar habitat to strain MD2411. In addition, the 100% 16S rRNA gene sequence similarity and phylogenetic positioning indicated that strain MD2411 and *Hapalosiphon welwitschii* UH IC-52-3 shared a species-level designation; thus, we identified MD2411 as *Hapalosiphon welwitschii* MD2411. On the other hand, Casamatta et al. (2020) mentioned the incorrect genus designations of several cyanobacteria within the family Hapalosiphonaceae in the literature and the NCBI Nucleotide database due to historically complicated taxonomy. Thus, based on the 16S rRNA phylogenetic relatedness of *Westiella intricata* UH HT-29-1 to these *Hapalosiphon* strains (Figure 2, cluster D), we suggested the inclusion of *Westiella intricata* UH HT-29-1 as a member of the genus *Hapalosiphon*.

Molecular analysis of the 16S rRNA gene sequences demonstrated that strain XN1103 shared 99.52% and 98.79% similarity with *Aulosira* sp. CENA272 and *Aulosira laxa* NIES-50, respectively (Table 2). In the phylogenetic tree, strain XN1103 formed a monophyletic clade (Figure 2, cluster C) with these strains with SH-aLRT/BS/BPP value of 92.7/99/1, in which *Aulosira laxa* NIES-50 was considered as a type strain of genus *Aulosira* (Hauer et al., 2014). Strain XN1103 had several morphometrical characteristics of vegetative cells and heterocysts similar to *Aulosira laxa* Kirchner ex Bornet & Flahault (1886–1888) (Lukesova et al., 2009), but it lacked the major features of *Aulosira* genus, such as intercalary heterocysts and apoheterocytic akinete formation. Additionally, we observed a new morphological characteristic in the *Aulosira* species; in XN1103, heterocyst always developed solely at the base of newly geminated trichome, and never appeared at other positions or in older trichomes (with necrotic vegetative cells). It is possible that this characteristic is shared by all species of *Aulosira* described previously (Shishido et al., 2020); however, this finding will need to be validated. Besides, the genus *Aulosira* was initially placed in the Microchaetaceae and was then transferred to Nostocaceae as mentioned by Hauer et al. (2014). The complicated taxonomic classification made it to be one of the genera with a limited number of species reported. Based on the typical habitat of rice paddy soil in the tropical region in which species of *Aulosira* was reported (Lukesova et al., 2009), the 16S rRNA gene sequence similarity [higher than 98.65% of the species threshold (Kim et al., 2014)], and the phylogenetic placement of 16S rRNA, we identified XN1103 as *Aulosira* species.

Strain NK1813 shared 99.28 % and 99.15% 16S rRNA gene sequence similarity to the strain *Desmonostoc danxiaense* CHAB5869 and *Desmonostoc persicum* SA14, respectively (Table 2). Although the dimension of vegetative cells, heterocysts, and the cells of the strain NK1813 were 1.6–2.5 times greater than those of strain *Desmonostoc danxiaense* CHAB5869 (Cai et al., 2018) and 1.4 times bigger than *Desmonostoc persicum* SA14 (Kabirnataj et al., 2020), they shared a prominent feature of oval, long akinetes chains (Figure 1R). The phylogenetic placement also supported the classification of strain NK1813 into the genus *Desmonostoc*, which included the type strain *Desmonostoc muscorum* Lukesova 1/87 (Hauer and Komarek, 2022), and together they formed a clade with SH-aLRT/BS/BPP values of 86.6/99/1 (Figure 2, cluster B). Due to the difference in the habitat of strain NK1813 (tropical region and hot climate) in comparison to the habitat with a low temperature of the strains CHAB5869 (rocky wall in Danxia mountain) and SA14 (soil, mediterranean-type climate), strain NK1813 was assigned to neither *Desmonostoc danxiaense* species nor *Desmonostoc persicum* species and was named as *Desmonostoc* sp. NK1813.

The short terminal branch lengths and high statistical supported value of 99.9/100/1 for species residing in cluster A (Figure 2) indicated that these strains were closely related, suggesting that their genus designation should be revised. Strain NS2000 shared 100% and 99.76% of 16S rRNA gene sequence similarity to that of *Nostoc* sp. NPA1 and *Nostoc* sp. PCC 9426, respectively. However, these three strains were distantly related to *Nostoc neudorfense* ARC8, which has been recently considered as the strain of the core *Nostoc* genus (Nostoc sensu stricto) (Hauer and Komarek, 2022). As reported in previous work (Pham et al., 2017), *Nostoc* sp. NPA1 belonged to the Nostoc sensu lato, which means it should be included in another genus rather than *Nostoc*. This clade also possessed *Trichormus azollae* Kom BAI/1983; however, three *Trichormus* strains included in this study showed a polyphyletic relationship (marked as ^*^ in Figure 2), indicating that the classification of these *Trichormus* strains should be revised. The misidentification of these *Trichormus* strains has been mentioned in the previous study by Rajaniemi et al. (2005), Komárek et al. (2013), and Saraf et al. (2018). On the other hand, strain NS2000 shared 98.04 % [higher than 94.5% of the genus threshold (Yarza et al., 2014)] of 16S rRNA gene sequence similarity to *Desikacharya soli* BHU2-PS. The phylogenetic analysis also supported the relatedness of strains NS2000 and NPA1 to *Desikacharya soli* BHU2-PS, suggesting that NS2000, as well as the *Nostoc* sp. PCC 9426 and *Trichormus azollae* Kom BAI/1983, may be considered as members of *Desikacharya* genus as recommended by Saraf et al. (2018). The morphological and morphometrical characteristics, and soil habitat of NS2000 (this study) and NPA1 (Pham et al., 2017) were consistent with those of *Desikacharya soli* BHU2-PS and *Desikacharya nostocoides* BHU1-PST in the study of Saraf et al. (2018). Thus, on the basis of the polyphasic approach, we assigned strain NS2000 as *Desikacharya* sp. and revised strain *Nostoc* sp. NPA1 as *Desikacharya* sp. NPA1.

In this study, *rbcL* gene sequences of five isolated strains and 31 reference sequences obtained from GenBank were used to build the phylogenetic tree (Figure 3). Unfortunately, most of the strains having the highest 16S rRNA gene sequence similarity to our investigated strains lacked published *rbcL* gene sequences, except for *Scytonema bilaspurene* 10C-PS. Therefore, the classification of four strains (MD2411, XN1103, NK1813, and NS2000) was not supported by the molecular data of *rbcL* gene regions due to the insufficient availability of *rbcL* sequences for type species or the closet species belonging to *Hapalosiphon, Aulosira, Desmonostoc*, and *Desikacharya* genera. The *rbcL* sequences for all strains generated in this study will contribute to the molecular database for further taxonomic studies of cyanobacteria. Despite the lack of a robust phylogeny based on *rbcL*, sequence data of 16S RNA and phylogenetic analysis based on ML and BI methods, together with morphological characteristics, were sufficient for the identification of these five strains in this study.

#### Cytotoxic activities

**Figure 4:**
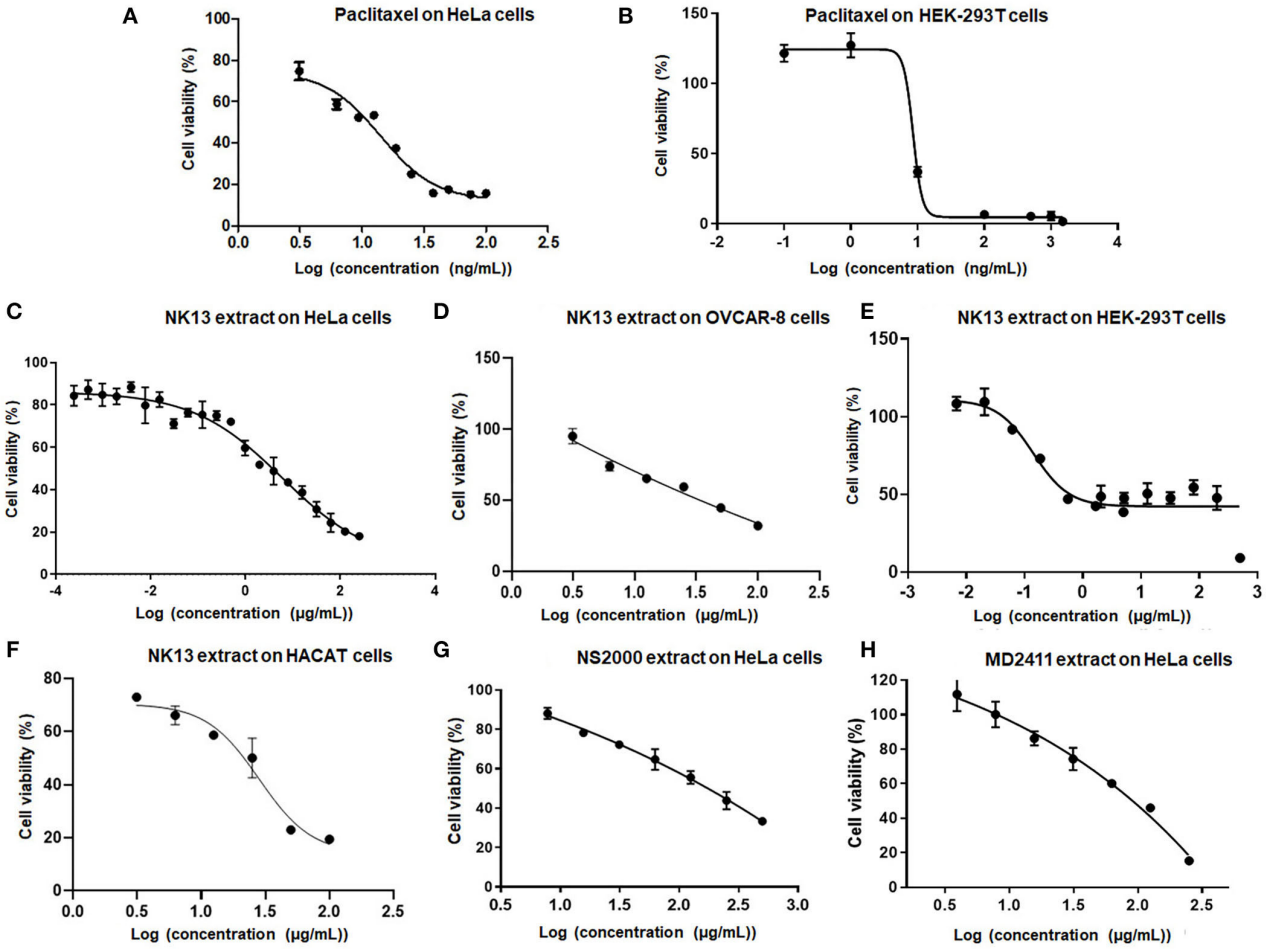
Dose-respond curves of paclitaxel **(A,B)** and several positive extracts **(C–H)** to human cells. HeLa, Human cervical adenocarcinoma cells; OVCAR-8, Human ovarian carcinoma cells; HEK-293T, Human embryonic kidney cells; HaCaT, Human epidermal keratinocyte cells. Each kind of extract was incubated with cells for 48 h, 4 parallels, *n* = 3.

The total extract of each strain was initially assessed for cytotoxicity against the HeLa cells. The results indicated that extracts from *Aulosira* sp. XN1103 and *Desmonostoc* sp. NK1813 were not active, but extracts from *Hapalosiphon welwitschii* MD2411, *Desikacharya* sp. NS2000, and *Scytonema bilaspurense* NK13 were able to inhibit the growth of HeLa cells. While the extracts from MD2411 and NS2000 showed marginal activities (Figures 4G,H) against Hela cells with IC_50_ of 91.07 and 165.7 μg/mL, respectively, the extract from NK13 was able to inhibit the growth of HeLa cells with an impressive IC_50_ value of 3.8 μg/mL (Table 4). The dose–response curve of the NK13 extract on HeLa cells (Figure 4C) had a similar pattern to that of paclitaxel which was used as the positive control (Figure 4A). The IC_50_ values of paclitaxel in our study (0.007–0.064 μg/mL) were in accordance with the previous studies of Sun et al. (2019) and Nguyen et al. (2021), ensuring the accuracy of cytotoxic tests. According to the US National Cancer Institute's criteria, an extract was considered as active if the IC_50_ value was below 20 μg/ml (Suffness and Pezzuto, 1991), which meant that the total extract of the strain *Scytonema bilaspurense* NK13 exhibited very high cytotoxicity.

**Table 4:** Cytotoxic activities of total extracts from five isolated cyanobacterial strains.

**No**	**Strain extracts**	**IC**_**50**_ **(**μ**g/mL) on cell lines**			
		**HeLa**	**OVCAR-8**	**HACAT**	**HEK-293T**
1	MD2411	91.07 ± 5.7	>100.0	>100.0	NT
2	NK13	3.8 ± 0.8	34.2 ± 2.7	21.6 ± 3.2	0.6 ± 0.5
3	NK1813	>200.0	NT	NT	>200.0
4	NS2000	165.7 ± 27.9	>100.0	>100.0	NT
5	XN1103	>200.0	NT	NT	NT
5	Paclitaxel (positive control)	0.012 (14.05 nM)	0.026 (30.44 nM)	0.064 (74.95 nM)	0.007 (8.55 nM)

NT, not tested; IC_50_ > 100 μg/mL, means no cytotoxic activity.

The cytotoxic activities of NK13 extract were confirmed on three other cell lines, including OVCAR-8 cells, HaCaT cells, and HEK-293T cells, with IC_50_ values of 34.2, 21.6, and 0.6 μg/mL, respectively (Table 4; Figures 4D–F). Interestingly, although the dose-respond curve of the NK13 extract on HEK-293T cells (Figure 4E) had a similar pattern to that of paclitaxel (Figure 4B), its concentrations in the range of 0.68–30.0 μg/mL caused the same effect on the cell growth, retaining 42% of cell viability. This phenomenon implied that the NK13 extract might inactivate mitotic processes of the HEK-293T cells but did not cause cell death at these concentrations.

The *Scytonema* strains have long been recognized as a potential source of biologically active compounds, and about 7.7 % of metabolite families have been reported from cyanobacteria till 2019 (Demay et al., 2019), with several well-documented compounds, such as scytovirin (antiviral activity), scytoscalarol (antibacterial activity), laxaphycin (antifungal activity), scyptolin A and B (enzyme inhibitory activity), mycosporine-like amino acid (MAA), and scytonemin (UV light absorption) (McFeeters et al., 2007; D'Agostino et al., 2016). Moreover, in the case of cytotoxic or anti-cancer activity, potent active compounds like scytophycin, tolytoxin, tubercidin, nostodione A, and scytonemide A and B were found in the members of *Scytonema* (Patterson et al., 1993; Patterson and Bolis, 1994; Shim et al., 2008). However, most of the strains exhibiting cytotoxic activities that have been discovered to date belonged to *S. saleyeriense, S. ocellatum, S. mirabile, S. burmanicum*, and *S. hofmannii* (Carmeli et al., 1990; Heinil et al., 2020). In this study, the potent cytotoxic activities of the tropical NK13 strain belonging to *S. bilaspurense* were reported for the first time.

## Conclusion

Recently, constructing systematics of cyanobacteria is a challenge because scientists worldwide must validate most of the morphological taxa in the past with new markers of polyphasic evaluation. It requires a large number of cyanobacteria strains and comprehensive data on each strain from all ecological niches on the Earth. In this study, we reported the isolation and characterization of five culturable cyanobacterial strains from the tropical region, which belong to three families in the Nostocales order. One strain belonged to Scytonemataceae family and was classified as *Scytonema bilaspurense* NK13, while one strain belonged to Hapalosiphonaceae and was classified as *Hapalosiphon welwitschii* MD2411. Three strains were assigned to the Nostocaceae family and designated as *Aulosira* sp. XN1103, *Desikacharya* sp. NS2000, and *Desmonostoc* sp. NK1813. Additionally, we have revised a group of strains NPA1, NPA6, APH5, APD3, APD4, APA4, APA5, and APA9 (KY818310) from Nostoc sensu lato in the previous study (Pham et al., 2017) and included in *Desikacharya* species. The cytotoxic evaluation of the total extract of each isolated strain led to the finding of one potent activity of extract from *Scytonema bilaspurense* NK13 against four cell lines. Thus, we appreciate this strain as a potential source of novel cytotoxic compounds and will be intensively investigated in the following study.

## Data availability statement

The datasets presented in this study can be found in online repositories. The names of the repository/repositories and accession number(s) can be found in the article/supplementary material.

## Author contributions

TTN: isolated strains, analyzed molecular data, and morphological characteristics. TD: constructed phylogenetic trees. B-LN, KK, and M-HD: cultivated cyanobacterial strains, prepared extracts, and performed cytotoxic tests. SN and T-HN: designed and performed molecular experiment. TLN and NT: interpreted cytotoxic data and edited manuscript. HP: conceived and designed research and wrote—original draft. All authors contributed to the article and approved the submitted version.

## Funding

This research is funded by the Vietnam National University, Hanoi (VNU) under project number QG.19.04.

## Conflict of interest

The authors declare that the research was conducted in the absence of any commercial or financial relationships that could be construed as a potential conflict of interest.

## Publisher's note

All claims expressed in this article are solely those of the authors and do not necessarily represent those of their affiliated organizations, or those of the publisher, the editors and the reviewers. Any product that may be evaluated in this article, or claim that may be made by its manufacturer, is not guaranteed or endorsed by the publisher.
